# Kindler epidermolysis bullosa associated with oral cancer in the buccal mucosa

**DOI:** 10.1016/j.jdcr.2022.06.005

**Published:** 2022-06-18

**Authors:** Eijiro Akasaka, Hajime Nakano, Daisuke Sawamura

**Affiliations:** Department of Dermatology, Hirosaki University Graduate School of Medicine, Hirosaki, Japan

**Keywords:** epidermolysis bullosa, Kindler, oral cancer, KEB, Kindler epidermolysis bullosa, SCC, squamous cell carcinoma

## Introduction

Kindler epidermolysis bullosa (KEB), also known as Kindler syndrome, is a rare autosomal recessive blistering skin disease caused by mutations in *FERMT1*, which encodes kindlin-1.^1^ Since Theresa Kindler described the first case of KEB in 1954,^2^ only approximately 250 cases have been reported worldwide. It is clinically characterized by skin blisters starting after birth, photosensitivity, progressive skin atrophy, and partial pseudosyndactyly.^3^ Patients are also prone to developing cutaneous squamous cell carcinoma (SCC).^4^ We herein present a case of KEB associated with oral cancer in the buccal mucosa; we show changes in typical clinical manifestations of KEB over time.

## Case report

**Fig 1:**
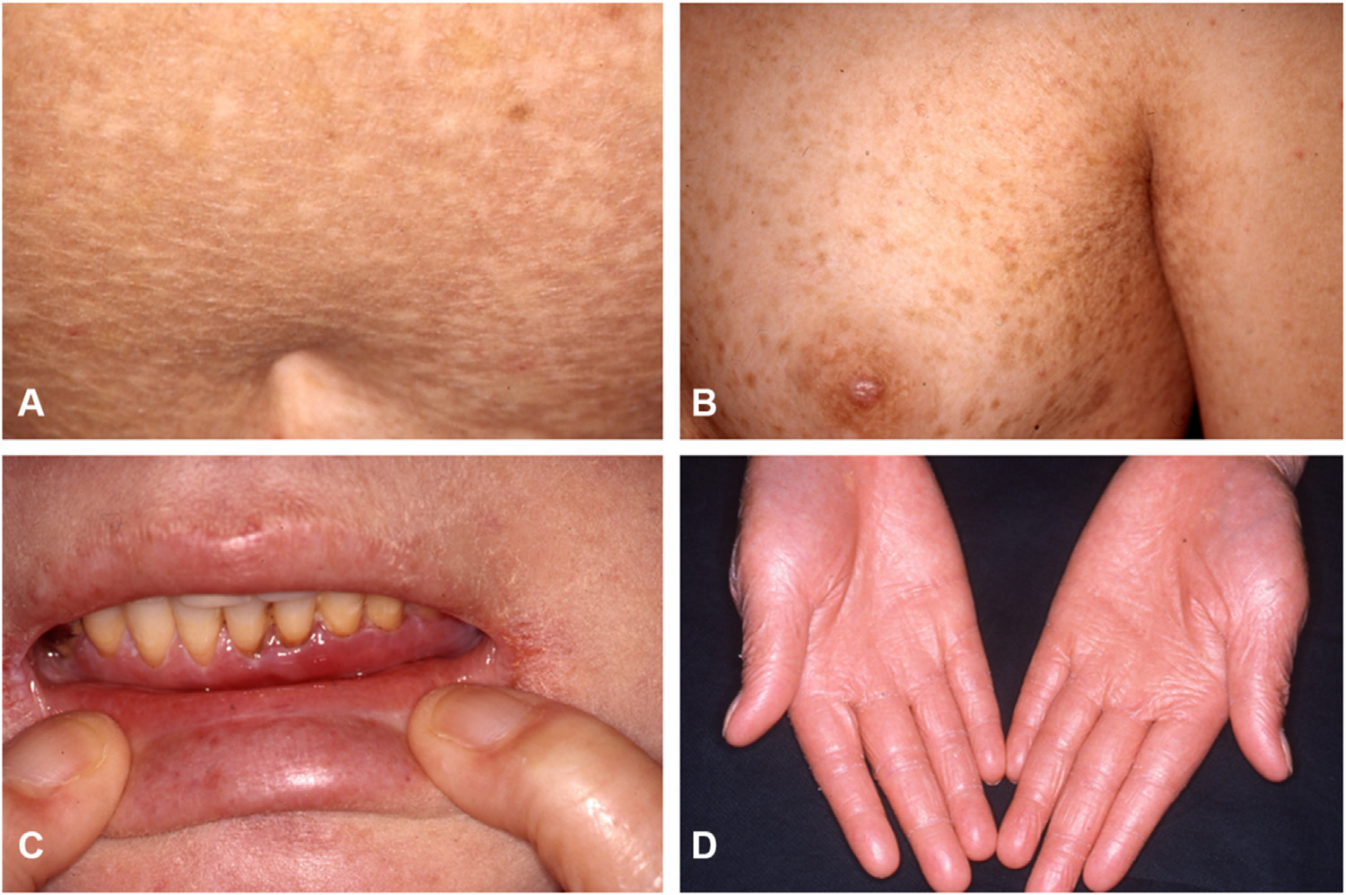
Clinical manifestations in 1983. **A,** Atrophic skin with depigmented spots on the abdomen. **B,** Mottled pigmentation on the chest. **C,** Atrophy and periodontitis on the gingiva. **D,** Mild hyperkeratosis on the palms.

A 24-year-old woman presented to our department with atrophic skin in 1983. She was born at term after an uneventful pregnancy as the second daughter of healthy nonconsanguineous parents. There was no known family history of skin diseases. She had presented with trauma-induced blisters on her extremities from birth. Blister formation ceased at age 12. However, photosensitivity and atrophic changes developed and slowly progressed. She presented with skin atrophy, mottled pigmentation, and depigmented spots all over her body (Fig 1, *A* and *B*); erosion and scars on her lips (Fig 1, *C*); atrophy and periodontitis on the gingiva (Fig 1, *C*); and mild hyperkeratosis on her palms and soles (Fig 1, *D*). These typical clinical features led to a diagnosis of KEB. As the pathogenic gene of KEB had not been identified at that time, mutational analysis was not performed. After confirmation of the diagnosis, medical follow-up was interrupted because of the patient’s personal reasons.

**Fig 2:**
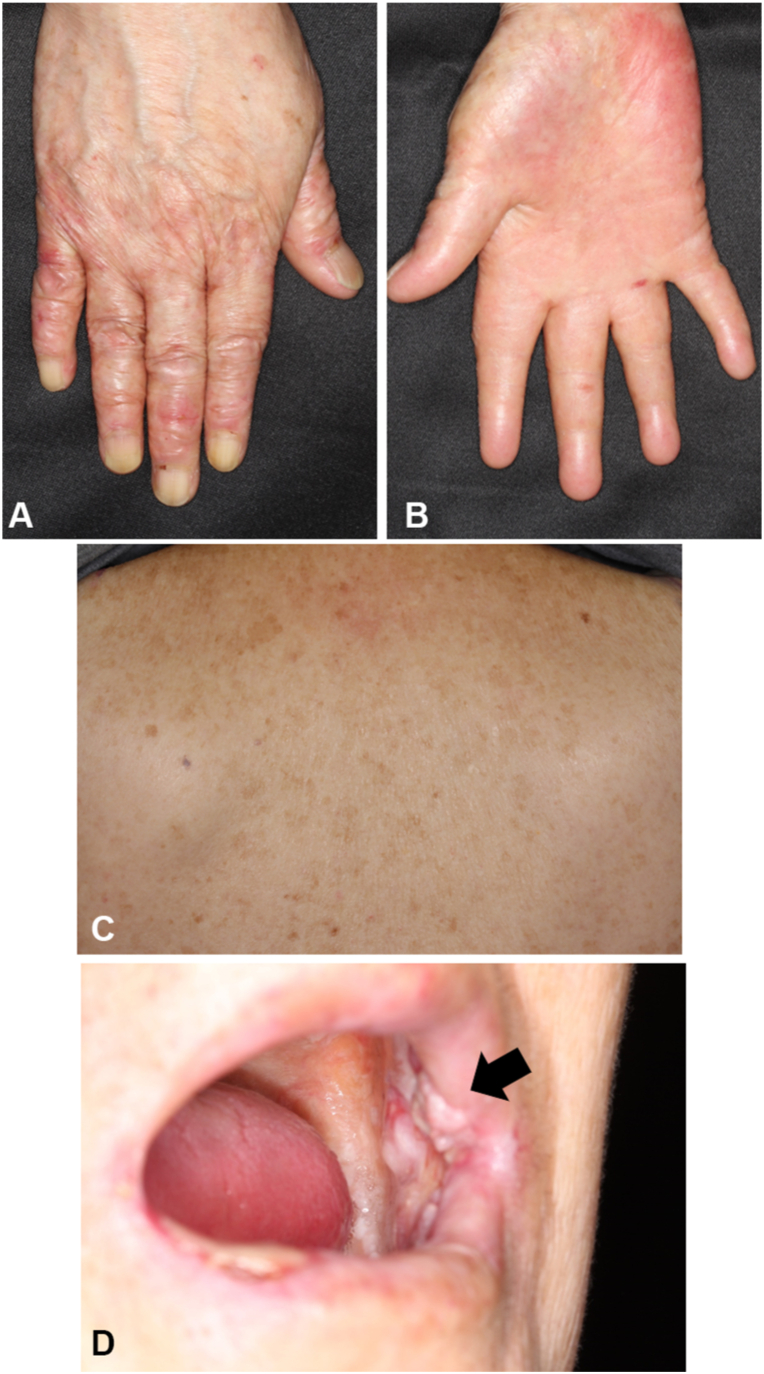
Clinical manifestations in 2021. **A,** Poikiloderma and cigarette paper-like appearance of the dorsal aspect of the right hand. **B,** Partial pseudosyndactyly with webbing between the fingers. **C,** Mottled pigmentation on the back. **D,** Oral cancer on the left buccal mucosa (*black arrow*). The patient was edentulous.

**Fig 3:**
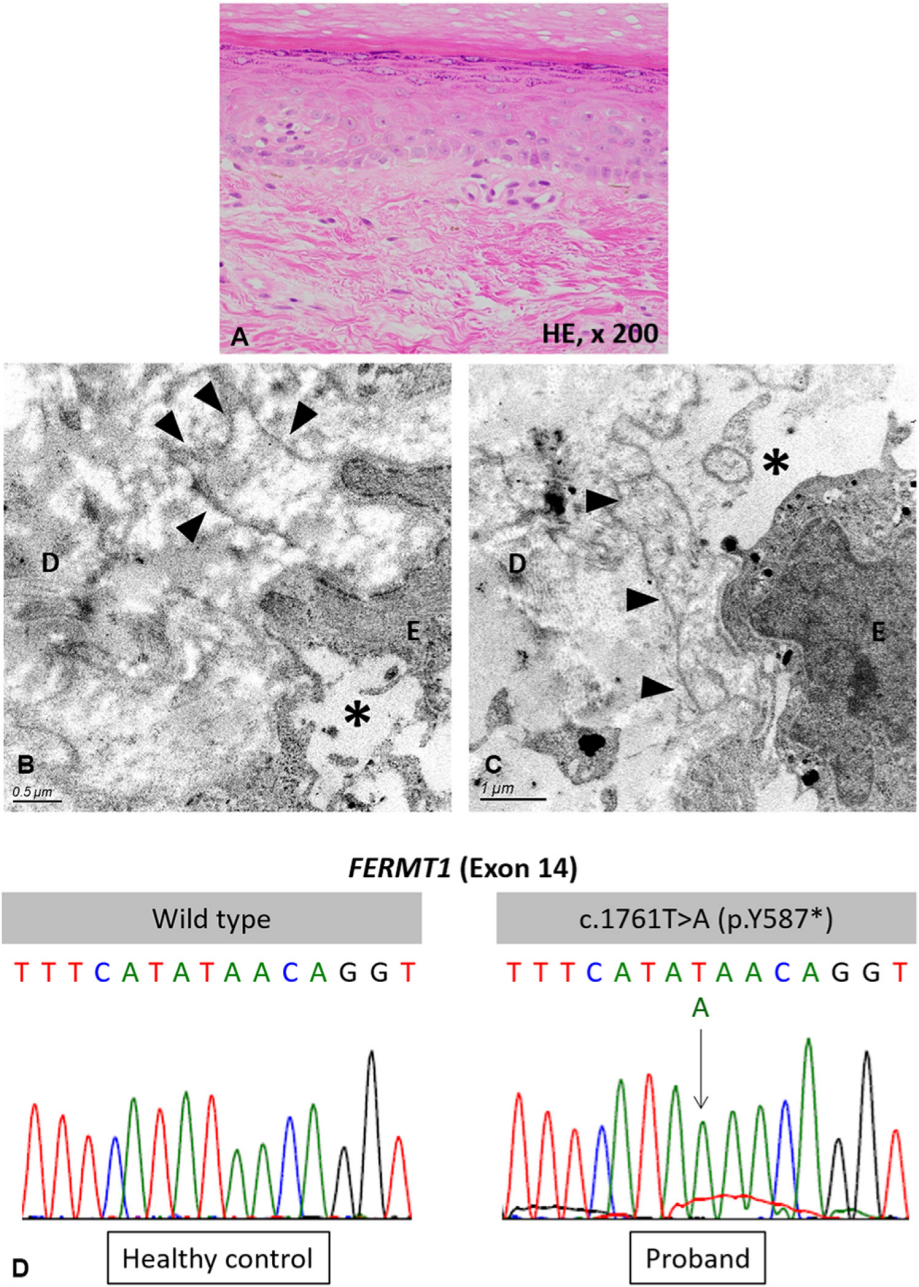
**A,** Histopathology of the skin. Flattened epidermis, vacuolar changes, and dilated capillaries in the papillary dermis (hematoxylin-eosinstain; original magnification: ×200). **B** and **C,** Transmission electron microscopy of the skin. Duplication of the lamina densa and tissue separation at the dermal-epidermal junction within the basal keratinocytes **(B)** and along the lamina lucida **(C)** (*E*, Epidermis; *D*, dermis; *arrowhead,* duplication of the lamina densa; *asterisk,* blister. Original magnification: ×20,000 (B) and ×30,000 (C), respectively). **(D)** Mutational analysis for *FERMT1*. The c.1761T>A (p.Y587∗) variant was detected in the homozygous state in the proband.

At age 61 (37 years later), she was hospitalized in the Department of Oral Surgery at our hospital to start chemoradiation therapy for oral cancer. The oral surgeon observed atrophic skin and consulted our department. Clinical examination revealed that the atrophic changes in her skin had become exacerbated over the past 37 years and now included poikiloderma and a cigarette paper-like appearance on the dorsum of the hands and feet (Fig 2, *A*). In addition, partial pseudosyndactyly with webbing between her fingers was also noted (Fig 2, *B*). Mottled pigmentation on her face, trunk, and proximal limbs as well as diffuse mild hyperkeratosis on her soles were still observed (Fig 2, *C*). All her teeth came out in her fourth decade. There was ulceration on her lips, atrophy of the oral mucosa, and a white ulcerative tumor on her left buccal mucosa (Fig 2, *D*). Histologic examination of a specimen obtained from the dorsum of her left hand demonstrated flattened epidermis with vacuolar changes and dilated capillaries at the papillary dermis (Fig 3, *A*). Transmission electron microscopy revealed duplication of the lamina densa and tissue separation at the dermal-epidermal junction within the basal keratinocytes and along the lamina lucida (Fig 3, *B* and *C*). After obtaining written informed consent, mutational analysis for *FERMT1* revealed a previously reported variant, c.1761T>A, in the homozygous state (Fig 3, *D*).^5^ The genetic test was approved by the ethics committee of the Hirosaki University Graduate School of Medicine (Approval no. 2020-16-5). The variant was predicted to result in a substitution of a tyrosine residue by a termination codon at amino acid 587, designated as p.Y587∗. Taken together, the diagnosis of KEB was confirmed.

## Discussion

To date, 91 distinct mutations of *FERMT1* have been identified, including 36 nonsense or missense mutations; 14 splice-site mutations; 31 small deletions, insertions, or indels; 8 gross deletions; and 2 other types of mutations (Human Gene Mutation Database [HGMD], http://www.hgmd.org). In the present study, mutational analysis of *FERMT1* revealed that the patient was likely to be homozygous for the previously reported nonsense c.1761T>A mutation.^5^ Unfortunately, her parents did not agree to genetic analysis. Therefore, we could not exclude the possibility that the patient harbors another pathogenic large deletion mutation in the entire sequence of *FERMT1* in a compound heterozygous state because haplotype analysis revealed she was homozygous for all detected single nucleotide polymorphisms in the gene (rs2273422, rs2273423, and rs2326719) (data not shown).

In this patient, we observed changes in clinical manifestations of KEB over time. Interestingly, poikiloderma, partial pseudosyndactyly, and interdigital webbing progressed with age. Although poikiloderma and mucocutaneous fibrosis are known to be later phenotypic features of KEB,^3^ the underlying mechanisms remain elusive. Mutations in *FERMT1* that induce premature termination codons, such as c.1761T>A (p.Y587∗) detected in this patient, lead to nonsense-mediated messenger ribonucleic acid decay and a lack of kindlin-1 protein in epidermal cells. Although kindlin-1 is not expressed in dermal fibroblasts,^6^ Heinemann et al^7^ demonstrated that kindlin-1–deficient keratinocytes respond to cellular stress by secreting IL-20 and IL-24, which directly induce type-I collagen expression in dermal fibroblasts. In addition, it has been reported that dermal fibroblasts obtained from patients with KEB express higher levels of profibrotic proteins such as tenascin-C, periostin, and transforming growth factor-β1 than normal dermal fibroblasts.^8^ Thus, the interaction between keratinocytes and fibroblasts via chronic inflammatory processes could play an important role in progressive skin fibrosis observed among patients with KEB.^7^

Furthermore, our patient developed oral cancer. Because kindlin-1 has been reported to suppress Wnt signaling, loss of kindlin-1 can be associated with tumorigenesis in several tissues via activation of canonical Wnt signaling.^9^ Guerrero-Aspizua et al^4^ reported that aggressive cutaneous SCC commonly develops in patients with KEB. The cumulative risk of SCC in patients with KEB increases with age, reaching 66.7% in patients > 60 years of age. They also noticed that the body distribution of SCC was concentrated in areas with high levels of inflammation, such as the hands and perioral areas. Despite the existence of a few reported cases of KEB with SCC on the oral mucosa,^10^ clinicians should be aware of the possibility of oral SCC in patients with KEB because the oral cavity is an area prone to inflammation.

In conclusion, we presented a case with KEB associated with oral cancer and documented progression of skin atrophy and fibrosis over time. This case report will provide useful clinical and genetic information for further understanding of this rare disease.

## Conflicts of interest

None disclosed.
